# Quantifying transmission of *Mycobacterium avium* subsp. *paratuberculosis* among group-housed dairy calves

**DOI:** 10.1186/s13567-019-0678-3

**Published:** 2019-08-20

**Authors:** Caroline S. Corbett, Mart C. M. de Jong, Karin Orsel, Jeroen De Buck, Herman W. Barkema

**Affiliations:** 1 Department of Production Animal Health, Faculty of Veterinary Medicine, University of Calgary, Calgary, AB T2N 4N1 Canada; 2 Quantitative Veterinary Epidemiology, Wageningen University, Droevendaalsesteeg 1, 6702 WD Wageningen, The Netherlands

## Abstract

Johne’s disease (JD) is a chronic enteritis caused by *Mycobacterium avium* subsp. *paratuberculosis* (MAP), with control primarily aimed at preventing new infections among calves. The aim of the current study was to quantify calf-to-calf transmission of MAP among penmates in an experimental trial. Newborn Holstein bull calves (*n* = 32) were allocated into pens of 4, with 2 inoculated (IN) calves and 2 calves that were contact exposed (CE). Calves were group-housed for 3 months, with frequent collection of fecal and blood samples and tissue collection after euthanasia. The basic reproduction ratio (R_0_) was estimated using a final size (FS) model with a susceptible-infected model, based on INF-γ ELISA and tissue culture followed by qPCR. In addition, the transmission rate parameter (β) for new shedding events was estimated using a general linearized method (GLM) model with a susceptible-infected-susceptible model based on culture, followed by qPCR, of fecal samples collected during group housing. The R_0_ was derived for IN and CE calves separately, due to a difference in susceptibility, as well as differences in duration of shedding events. Based on the FS model, interferon-γ results from blood samples resulted in a R_0_^IG^ of 0.90 (0.24, 2.59) and tissue culture resulted in a R_0_^T^ of 1.36 (0.45, 3.94). Based on the GLM model, the R_0_ for CE calves to begin shedding (R_0_^CE^) was 3.24 (1.14, 7.41). We concluded that transmission of MAP infection between penmates occurred and that transmission among calves may be an important cause of persistent MAP infection on dairy farms that is currently uncontrolled for in current JD control programs.

## Introduction

*Mycobacterium avium* subsp. *paratuberculosis* (MAP) is the causative agent of Johne’s disease (JD), a chronic enteritis primarily affecting ruminants and causing substantial losses to dairy industries worldwide [1–3]. There is currently no treatment, cure, or vaccine for the prevention of MAP infection in cattle; therefore, control is primarily based on preventing transmission of MAP and reducing new infections within the herd [2, 4–6].

The primary route of MAP transmission is fecal–oral through contaminated feed, milk, water and the environment caused by infectious animals intermittingly shedding MAP in their feces [7–9]. To decrease transmission from cows to calves and limit exposure of young stock to MAP, calves are removed from adult cows as soon as possible after birth and placed in calf barns or pens [10, 11]; however, calves up to 1 year of age have demonstrated susceptibility to MAP infection and calves can begin fecal shedding of MAP bacteria as early as 2 weeks after exposure [12–15]. Therefore, separating calves from cows and subsequent group housing may not be an effective method for prevention of new infections in young stock. Despite evidence that calf-to-calf transmission can occur [14, 16], findings regarding implications and impact of this route of transmission on control within herds were inconsistent [16–20]. Additionally, statistical analyses of data from 21 MAP infected farms suggested that transmission among calves was necessary to explain observed patterns of transmission within a herd [21].

Susceptible-infected-susceptible (SIS) models are modified from susceptible-infected-recovered (SIR) models; both have been used to model transmission of pathogens in a population [22–25]. Transmission of pathogens is quantified using the basic reproduction ratio (R_0_), the average number of new cases caused by 1 typically infectious individual introduced into a completely susceptible population [26, 27]. The threshold at which an outbreak can occur is when the R_0_ value is > 1, whereas an infection is certain to fade out in a population if R_0_ is < 1 [26]. Transmission dynamics for MAP infection are notoriously difficult to determine, due to long incubation, latently infected animals, variability of diagnostic tests and lack of long-term randomized control studies [28]. Deterministic and stochastic mathematical modelling techniques have been used to investigate MAP spread and transmission parameters [21, 29, 30], assess impact of varying infectious animals, as well as varying levels of environmental contamination [9, 31, 32], economic impacts of disease [33] and to determine effectiveness of control programs and interventions on spread and control of this disease [34, 35]. Due to complications regarding MAP infection, these models all rely on educated guesses regarding infectivity and susceptibility of animals in a herd, as well as impacts of various transmission routes. Increasing knowledge regarding transmission within a herd will enhance understanding of disease maintenance and spread within a herd and enable control programs to be optimised to better manage spread of infection [36]. Although transmission among calves is possible, sufficient quantitative information regarding amount of transmission is lacking. The aim of the current study was, therefore, to quantify calf-to-calf transmission of MAP infection among penmates and estimate R_0_ for calves identified as infected, or infectious, based on various diagnostic tests.

## Materials and methods

### Experimental design

**Figure 1:**
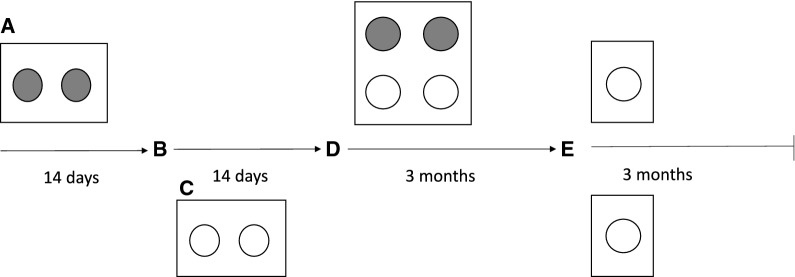
**Experimental design for quantification of** ***Mycobacterium avium*** **subsp.** ***paratuberculosis*** **among group-housed calves.** One of 7 experimental pens represented, containing 2 inoculated (IN) calves (grey circles) and 2 contact exposed (CE) calves (white circles). a) 2 newborn calves entered the barn and were housed in a clean environment for 2 weeks; b) IN calves were inoculated with 5 × 10^8^ CFU’s of MAP at 2 weeks of age; c) 2 weeks of IN housing to allow pass-through of inoculum. During this time, 2 newborn calves entered the barn and were designated as CE and housed apart from IN calves; d) first day of group housing. IN and CE calves were co-mingled in a clean pen and remained group-housed for 3 months; e) IN calves removed for necropsy, CE individually housed for additional 3 months.

Study design, calf collection and sample collection were as described [14]. Briefly, 32 newborn Holstein–Friesian bull calves were purchased from 13 Alberta (Canada) dairy farms that had tested negative for MAP for at least 4 years based on culture of environmental samples and milk ELISA or individual fecal sampling of the herd. Calves were assigned to infection status and pen based on birth order and entry into the Biosecurity Level-2 facility, with 7 experimental group pens each consisting of 2 inoculated calves (IN) and 2 recipient contact-exposed calves (CE) (Figure 1). The last 4 calves to enter the barn were designated controls. At time of group housing, IN calves were on average 4 weeks of age, whereas CE calves were 2 weeks of age in each experimental pen. Calves were group-housed in pens of 4 (2 IN, 2 CE) for 3 months after inoculation. After 3 months of group housing, IN calves were euthanized and remaining calves in experimental pens were individually housed (87 days after 1st day of group housing) for an additional 3 mo. At the end of the trial, all remaining calves were euthanized for tissue sampling. Control calves were group-housed for the entirety of the study. All protocols and the experimental design were approved by the University of Calgary Veterinary Sciences Animal Care Committee (protocol AC14-0168).

### Inoculum

Inoculum preparation was as described [14]. A virulent strain from a clinical case of JD (cow 69) in Alberta was used for inoculation. Two calves in each experimental pen were inoculated with an oral dose of 2.5 × 10^8^ CFU on 2 consecutive days at 2 weeks of age. To ensure shedding, this dose was higher than the minimum dose of 10^6^ recommended for experimental infection and detection in tissues [37, 38]. Concurrently, this dose was also lower than previous experimental trials that used “high” doses greater than 10^9^ [39], which are unlikely to be comparable to natural infectious doses and ensuing shedding. Following inoculation, calves remained individually housed for 2 weeks to allow passive shedding of the inoculum to cease before being group-housed with recipient calves.

### Sampling

Fecal, blood and tissue samples were all collected as described [14]. Briefly, individual fecal samples were collected three times/week for 3 months, starting 1 day after the onset of group housing. Samples were stored at 4 °C and processed within 7 days after collection. All fecal samples were processed using modified TREK ESP II culture media (TREK para-JEM^®^; TREK Diagnostic Systems, Cleveland, OH, USA) as described [12, 14]. Following processing, fecal samples were incubated at 37 °C for 49 days.

Blood samples were collected weekly from the jugular vein, alternating between sides. Within 2 h after collection, samples were transported to the laboratory (in an insulated box with hot water bottles at 25–35 °C).

After 3 months of group housing (i.e., 4 months of age), IN calves were euthanized by intravenous injection of barbiturates (Euthanyl Forte^®^, DIN 00241326, Bimeda-MTC Animal Health Inc., Cambridge, ON, Canada), necropsied and tissue samples collected. After an additional 3 months of being housed individually, CE calves were euthanized, necropsied and tissues collected. Thirteen tissue samples were collected from each calf, including sections of the ileum, jejunum, duodenum, all associated lymph nodes and spleen. After transportation to the laboratory, samples were processed immediately for culture, as described [14]. Briefly, 2.5 g of tissue was dissociated and disinfected prior to incubation within paraJEM^®^ culture bottles and incubated at 37 °C for 49 days.

### Detection of MAP

Following liquid culture of fecal and tissue samples for 49 days, DNA was extracted as described [14, 40]. A duplex qPCR was performed targeting the MAP-specific F57 region and an internal amplification control (IAC) with primers, probes and IAC sequences identical to those described [41]. Samples were considered positive if the cycle threshold (CT) value was < 40.

### Detection of an immune response

Following transportation to the laboratory, blood samples were processed for detection of IFN-γ release, as described [14, 42]. Each whole-blood sample was treated with 100 μL avium Purified Protein Derivative (aPPD; 0.3 mg/mL; Canadian Food Inspection Agency, Ottawa, ON, Canada), 100 μL of pokeweed mitogen (positive stimulation control; 0.3 mg/mL; Sigma-Aldrich Canada Co., Oakville, ON, Canada) and 100 μL sterile PBS (negative stimulation control). Following overnight incubation at 37 °C and centrifugation serum was collected and assayed using the BOVIGAM^®^ sandwich ELISA (Prionics, La Vista, NE, USA). The %IFN-γ was calculated as follows: [(PPD Johnin-negative assay control)/(positive–negative assay control)] × 100 [42, 43].

### Quantification of MAP transmission

#### GLM and FS model

The transmission rate parameter β represents the average number of new infections in a fully susceptible population caused by 1 typically infectious animal per unit of time [27]. The basic reproduction ratio (R_0_) represents the average number of new infections in a totally susceptible population caused by 1 typically infectious calf during its infectious period and was estimated using 2 approaches. The generalized linear model (GLM) was based on an SIS model (infectious status determined during the experiment based on fecal shedding) to quantify transmission parameter β (from which R_0_ can be derived). The GLM model is based on fecal shedding, which can only identify infectious animals and not infected animals. Therefore, it is a model quantifying the transmission parameter for a calf to shed MAP. The final size (FS) models were based on an SI model (infection status determined at end point, with no animals recovering) to estimate R_0_ directly [23], in which infection dynamics were based on number of recipient animals, i.e. susceptible for the FS, or not infectious for the GLM (S), infectious for the GLM (shedding), or infected for the FS (I) and total number of animals (N). Thus, in a classical model for the GLM analysis, the rate at which susceptible animals (S) are infected is given by βSI/N and the probability of a single susceptible calf becoming infected during a period $\Delta {\text{t}}$ is:

$${\text{p}} = 1 - {\text{e}}^{{ -\upbeta_{\text{T}} \times \frac{{{\text{I}}_{\text{t}} }}{{{\text{N}}_{\text{t}} }} \times \Delta {\text{t}}}}$$

where $\upbeta_{\text{T}}$ is the total transmission rate parameter [27]. Data were analyzed using STATA 11 (StataCorp LP, College Station, TX, USA). All confidence intervals refer to 95% confidence intervals.

#### General linearized model (GLM)

The GLM used a binomial distribution, with the dependent variable being number of new cases, which in this case are shedding events (C) and total number of susceptible (S) calves as the binomial total. The analysis was done with a complementary log–log (cloglog) link function, a binomial error term and an offset explained below [27, 44].

The expression for the GLM was:

$${\text{cloglog E}}\left( {\frac{{\text{C}}}{{\text{S}}}} \right) = \log\upbeta_{{\text{T}}_{\text{t}}} + { \log }\left( {\frac{{{{\text{MAP}}}_{{{\text{T}}}_{{\text{t}}}} }}{{{\text{N}}}_{{\text{t}}}} \cdot \Delta {{\text{t}}}} \right),$$

where $\log\upbeta_{{{\text{T}}_{\text{t}} }}$ is the intercept and thus the logarithm of the transmission parameter is the (intercept) regression coefficient and ${ \log }\left( {\frac{{{\text{MAP}}_{{{\text{T}}_{\text{t}} }} }}{{{\text{N}}_{\text{t}} }} \cdot \Delta {\text{t}}} \right)$ is the offset variable; ${\text{E}}\left( {\frac{{\text{C}}}{{\text{S}}}} \right)$ = expected number of cases (C) during the infectious interval (t, t + $\Delta {\text{t}}$) divided by the number of susceptible individuals (S) at the start of the time interval (i.e. at t); β_T_ = total transmission rate parameter; MAP_T_ = total amount of infectious material (animals and environment), also at the start of the time interval (t); Δt = duration of the time interval; and N_T_ = total number of animals at the start of the time interval (t) as this measures the size of the area given that density is constant [45]. Infectious material in the environment, or shed by an infectious calf (I), were assumed to be equally infectious as the primary route of MAP transmission is fecal–oral. Thus on any given day, transmission occurs due to shedding calves and the amount of bacteria in the environment due to prior shedding in the pen.

Note that the β_T_ is the total transmission rate parameter. Due to several potential routes of transmission, different transmission effects may contribute to β_T_ and hence different transmission rate parameters can be estimated depending on the population composition, as discussed below. Additionally, infectious calves can either cause new infections through direct contact (I_IN_ or I_CE_) with susceptible calves, or through environmental contamination caused by infectious calves (E_IN_ or E_CE_), as discussed below. Therefore,

$${\text{MAP}}_{\text{T}} = {\text{MAP}}_{\text{IN}} + {\text{MAP}}_{\text{CE}}$$

$${\text{MAP}}_{\text{IN}} = {\text{I}}_{\text{IN}} + {\text{E}}_{\text{IN}}$$

$${\text{MAP}}_{\text{CE}} = {\text{I}}_{\text{CE}} + {\text{E}}_{\text{CE}}$$

Note the definition of MAP_T_ implies the following:

$${\text{MAP}}_{\text{T}} = {\text{I}}_{\text{IN}} + {\text{E}}_{\text{IN}} + {\text{I}}_{\text{CE}} + {\text{E}}_{\text{CE}}$$

The GLM analysis is based on interval data and was therefore based on fecal samples collected over the course of the trial using the SIS model. At any given time interval within each pen, infectious (I) individuals were those with MAP-positive fecal results, susceptible individuals (S) had MAP-negative fecal results and new cases (C) were calves with negative fecal culture results at the previous observation point that became infectious (I) in the current time interval. Changes in individual status were observed for each new time interval within each of the 7 experimental pens, either 2 or 3 days apart.

All time intervals where a pen had 0 susceptible (S) calves were removed from the analysis (*n* = 9). The 95% confidence interval (CI) of the estimated β parameters was calculated using the standard error of the mean of log β.

#### Estimation of MAP transmission through the environment: β_E_

Environmental contamination caused by fecal shedding calves was included in the model as a route of transmission for infection (expressed as parameter β_E_). On the first day of group housing, calves were introduced to a clean, MAP-free environment. Therefore, environmental contamination was the result of MAP accumulating in the environment from either IN or CE calves shedding. We assumed that environmental contamination on a specific day (E_t_) resulted from accumulation of MAP in the environment by infectious individuals (either IN or CE) on the previous day (I_(t−1)_), as well as the remaining exposed MAP in the environment from all anterior shedding events prior to t − 1 (E_t−1_). Environmental contamination caused by previous shedding events were discounted by the concealment rate (γ) for every day after the shedding event. Concealment and resulting exposure rate of MAP were estimated as described, based on survival rate definitions [22] and accounting for potential differences in contamination caused by IN or CE calves. Briefly, E_T_ was calculated as the sum of environmental contamination caused by IN (E_IN_) and CE (E_CE_) calves, ${\text{E}}_{\text{T}} = {\text{E}}_{\text{IN}} + {\text{E}}_{\text{CE}}$.

The same concealment rate (γ) was applied to both E_IN_ and E_CE_ and was calculated based on the assumption that fecal–oral transmission from the environment will be the same as fecal–oral transmission from an infectious pen mate on the same day [22]. Total oral transmission (I_T_ = I_IN_ + I_CE_) directly to other calves within the same time period (t) as the infectious material is shed, is basically the same as will occur from the equivalent but diminished amount of material in subsequent time periods. MAP remains viable in the environment for extended periods of time and the infectious dose is not known; it can, therefore, be assumed that a calf picking MAP up directly from fecal shedding of a pen mate (I) or from the environmental contamination (E) would have equal probability of getting infected. However, over time, MAP will be concealed (diminished) in the environment and exposure of the other animals decreased, resulting in lower transmission [22]. Using new cases (C) as the result of environmental contamination (new cases that occurred following shedding in the pen at previous sampling point (t − 1)) for all time intervals in all pens, we estimated γ to be equal 0.1422 day^−1^ as described [22]. A sensitivity analysis in which concealment rate was increased to 0.5 and decreased to 0.001 was performed to ensure the best estimate was made for the model and was in agreement with current literature [46]. Time intervals (t) in the current study were either 2 or 3 days long; therefore, the exposure (survival) rate (σ) was calculated based on the following equation: $\sigma = e^{ - \gamma *\Delta t}$ with ∆t = 2 or 3 days. E_T_ for period t was then calculated as follows:

$${\text{E}}_{{{\text{T}}_{\text{t}} }} = \left( {\upsigma_{{\left( {{\text{t}} - 1,{\text{t}}} \right)}} \left( {{\text{I}}_{{IN\left( {t - 1} \right)}} } \right)} \right) + \left( {\upsigma_{{\left( {{\text{t}} - 1,{\text{t}}} \right)}} \left( {{\text{I}}_{{{\text{CE }}\left( {{\text{t}} - 1} \right)}} } \right)} \right) +\upsigma_{{\left( {{\text{t}} - 1,{\text{t}}} \right)}} E_{{T \left( {t - 1} \right)}} ,$$

where $\left( {\upsigma_{{\left( {{\text{t}} - 1,{\text{t}}} \right)}} \left( {{\text{I}}_{{{\text{IN }}\left( {{\text{t}} - 1} \right)}} } \right)} \right)$ is the environmental contamination caused by direct shedding of infectious material of IN calves in the time interval prior to t; $\left( {\upsigma_{{\left( {{\text{t}} - 1,{\text{t}}} \right)}} \left( {{\text{I}}_{{{\text{CE }}\left( {{\text{t}} - 1} \right)}} } \right)} \right)$ is the environmental contamination caused by direct shedding of infectious (I) CE calves in the time interval period prior to time t; and $E_{{T \left( {t - 1} \right)}}$ is the amount of environmental accumulation resulting from all previous shedding events leading up to a time interval i.e. up to t-1 (accounting for the concealment rate over time). Total environmental contamination (E_T_) for a specific period (t) was calculated based on the number of calves shedding in the time interval immediately prior to t (t−1) and any environmental contamination already in the prior interval was added to account for the accumulation effect.

#### Transmission rate parameters

The transmission rate parameter β_T_ is dependent on the population composition in a pen and represents all transmission that could occur between the pen mates in a group housing pen. Susceptibility and infectivity among calves can be variable, with part of that variation attributed to observable differences, e.g. inoculation status. These differences in shedding patterns between penmates influence the transmission rate parameter β, as not all calves can be assumed to have the same susceptibility and infectivity. All calves were either inoculated (IN) or contact exposed (CE) and this dichotomy can influence susceptibility to begin and to continue shedding, as well as impact how infectious a calf is i.e. propensity to cause shedding in other calves by the shedding calf. To differentiate susceptibility status of IN and CE calves, we used an explanatory dummy variable (INO) classified as 0 or 1 for the recipient animal to be either inoculated (INO = 1) or contact infected (INO = 0). Additionally, the fraction of infectivity for either IN or CE can be calculated based on direct fecal shedding and contamination in the environment. For example, the expression for the fraction of infectivity caused by infectious IN calves in one time interval is:

$${\text{f}}_{\text{IN}} = \frac{{{\text{I}}_{\text{IN}} + {\text{E}}_{\text{IN}} }}{{{\text{I}}_{\text{IN}} + {\text{E}}_{\text{IN}} + {\text{I}}_{\text{CE}} + {\text{E}}_{\text{CE}} }}$$

where ${\text{I}}_{\text{IN}} + {\text{E}}_{\text{IN}}$ is the total infectivity caused by IN calves (MAP_IN_) and ${\text{I}}_{\text{IN}} + {\text{E}}_{\text{IN}} + {\text{I}}_{\text{CE}} + {\text{E}}_{\text{CE}}$ is total infectivity caused by all calves (MAP_T_).

Additionally, we can test whether transmission by direct shedding in the current period, or in subsequent periods due to environmental contamination, are the same. These comparisons are based on calculating the fraction of the total environmental infectivity where ${\text{f}}_{\text{E}} = \frac{{{\text{E}}_{\text{IN}} + {\text{E}}_{\text{CE}} }}{{{\text{MAP}}_{\text{T}} }}$ and ${\text{E}}_{\text{IN}} + {\text{E}}_{\text{CE}}$ is equal to total infectivity in the environment (E_T_).

Yet another explanatory variable we added was the day since the start of group housing (day). This variable has the same value for all calves in the pen for each observed interval and thus the corresponding regression coefficient contained both susceptibility and infectivity effects. When main effects were significant, potential interactions were also examined.

**Figure 2:**
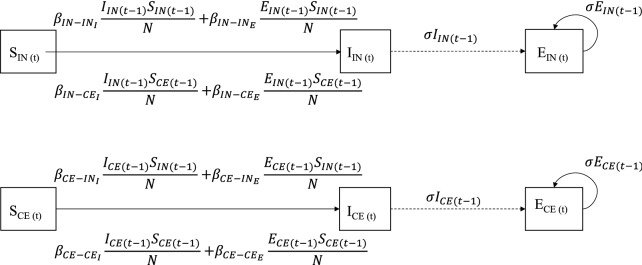
**The GLM model for 1 time interval.** The transmission rate parameters depend on the number of infectious IN and CE calves (I_IN_ and I_CE_, respectively) and/or on the amount of MAP in the environment caused by IN or CE shedding (E_IN_ and E_CE_, respectively). E depends on MAP secretion in current time interval from the infectious calves (either IN or CE) and amount of MAP remaining in the environment from excretion by the infected calves on previous days (t-1) weighted by σ.

Thus, based on the above, the following transmission route parameters were quantified using GLM (Figure 2), due to transmission caused by: direct contact between IN calves ($\beta_{{IN\_IN_{I} }}$), direct contact from IN calves to CE calves ($\beta_{{IN\_CE_{I} }}$), environmental contamination between IN calves ($\beta_{{IN\_IN_{E} }}$), environmental contamination from INto CE calves ($\beta_{{IN\_CE_{E} }}$), direct contact between CE calves ($\beta_{{CE\_CE_{I} }}$), direct contact from CE calves to IN calves ($\beta_{{CE\_IN_{I} }}$), environmental contamination between CE calves ($\beta_{{CE\_CE_{E} }}$) and environmental contamination from CE calves to IN calves ($\beta_{{CE\_IN_{E} }}$), where $\beta_{{IN\_IN_{I} }} ,\beta_{{IN\_IN_{E} }} ,\beta_{{IN\_CE_{E} }} ,\beta_{{CE\_CE_{I} }} ,\beta_{{CE\_CE_{E} }} ,\beta_{{CE\_IN_{I} , }} and \beta_{{CE\_IN_{E} }}$ and all are based on regression coefficients in the following equation:

$${\text{cloglog E}}\left( {\frac{{\text{C}}}{{\text{S}}}} \right) = {\text{C}}0 + {\text{C}}1 * {\text{INO}} + {\text{C}}2 * {\text{day}} + {\text{C}}3 *\frac{{{{\text{I}}}_{{\text{IN}}} + {{\text{E}}}_{{\text{IN}}} }}{{{{\text{MAP}}}_{{\text{T}}} }} + {\text{C}}4 \frac{{{{\text{E}}}_{{\text{IN}}} + {{\text{E}}}_{{\text{CE}}} }}{{{{\text{MAP}}}_{{\text{T}}} }} + {{\text{C}}}5 {{\text{INO*day}}} + \log \left( {\frac{{{{\text{MAP}}}_{{\text{T}}} }}{{{\text{N}}}_{{\text{t}}}} \cdot \Delta {\text{t}}} \right).$$

The regression coefficients can be interpreted as follows: C0 is the intercept and is the log of the transmission rate parameter of the reference that is direct transmission from contact-exposed calves (that have begun fecal shedding) to other contact-exposed calves at day 0 of the experiment; C1 is the extra effect of the recipient being of the inoculated type (susceptibility to begin shedding); C2 is the extra effect on transmission with days since the start of infection (susceptibility and infectivity); C3 is the extra effect on infectivity if the infectious calf is of the inoculated type; C4 is the extra infectivity of a unit of infectious material arriving at the recipient through the environment; and C5 is the effect of the interaction of susceptibility of an inoculated recipient and the day since the start of the experiment.

From this, transmission rate parameters can be derived as follows (assuming that all regression coefficients were significantly different from zero):

$\upbeta_{{IN\_IN_{I} }} = e^{C0 + C1 + C2 + C3 + C5}$ is the transmission rate parameter from an infectious IN calf to a susceptible IN calf through direct contact;

$\upbeta_{{IN\_IN_{E} }} = e^{C0 + C1 + C2 + C3 + C4 + C5}$ is the transmission rate parameter from an infectious IN calf to a susceptible IN calf through the environment;

$\upbeta_{{CE\_CE_{I} }} = e^{C0 + C2}$ is the transmission rate parameter from an infectious CE calf to a susceptible CE calf through direct contact;

$\upbeta_{{CE\_CE_{E} }} = e^{C0 + C2 + C4}$ is the transmission rate parameter from an infectious CE calf to a susceptible CE calf through the environment;

$\upbeta_{{IN\_CE_{I} }} = e^{C0 + C2 + C3}$ is the transmission rate from an infectious IN calf to a susceptible CE calf through direct contact;

$\upbeta_{{IN\_CE_{E} }} = e^{C0 + C2 + C3 + C4}$ is the transmission rate parameter from an infectious INto a susceptible CE calf through the environment;

$\upbeta_{{CE\_IN_{I} }} = e^{C0 + C1 + C2 + C5}$ is the transmission rate parameter from an infectious CE to a susceptible IN calf through direct transmission; and

$\upbeta_{{CE\_IN_{E} }} = e^{C0 + C1 + C2 + C4 + C5}$ is the transmission rate parameter from an infectious CE to a susceptible CE calf through the environment.

Regression coefficients were tested for being different from zero. For regression coefficients not significantly different from zero (*p* > 0.05), the corresponding explanatory variable was dropped from the model unless it led to confounding (> 25% change in the regression coefficient of the other explanatory variables). Interaction terms were only calculated when both main effects were statistically significant.

#### Infectious periods: T_IN_ and T_CE_

Infectious periods were calculated based on duration of shedding events for IN and CE calves as described [14]. The first time a calf tested MAP-positive in fecal samples was considered day 1 of its infectious period. The infectious period then would end if the next sample was negative, or continue until a negative sample was detected. A shedding event was defined as continuous streak of positive fecal samples and ranged from 2 days (1 positive fecal sample) up to 54 days (23 consecutive positive fecal samples) during the 90+ days of group housing.

Mean duration of infectious period for IN calves (T_IN_) and CE calves (T_CE_) was calculated as the average length of shedding events for IN calves or CE calves, respectively. The 95% confidence intervals (CI) were calculated using the standard variation of the mean of log T_IN_ and log T_CE_ for IN calves and CE calves, respectively.

#### Using the GLM: R_0_^CE^ and R_0_^IN^

The R_0_ can be estimated by multiplying the rate of transmission (β) by the infectious period (T). The concealment rate by the environment due to new bedding being added and infectious material being covered was also taken into consideration using the variable $\upsigma$, thus $(1 -\upsigma)^{ - 1}$ represents the total exposure rate of MAP in the environment for each day of the infectious period T. The equation for estimating R_0_ is:

$${\text{R}}_{0} = * {\text{T*}}(1 -\upsigma)^{ - 1}$$

The 95% CI of R_0_ was calculated using the variance and regression constant of the GLM results (log β) and the variance and the average of the logarithm of the infectious period T. R_0_ is calculated separately for CE and IN, due to differences in infectiousness and susceptibility to begin shedding. Therefore, the estimated basic reproduction value for CE calves is R_0_^CE^ and R_0_^IN^ for inoculated calves.

#### Final size (FS) model: R_0_^T^, R_0_^IG^

The FS model was based on the SI model, which uses the total number of susceptible animals remaining at the end of the experiment [23]. Two FS models were used to account for varying definitions of MAP infection. In the first FS model, infected (I) calves were counted as those with at least one culture-positive tissue sample by the end of the experiment, and remaining susceptible (S) calves as those with no positive tissue sample. R_0_^T^ was the estimate of the number of new tissue-positive infections that would result from the introduction of one tissue-positive calf to the susceptible population. In the second FS model, infected (I) calves were those with at least 1 positive INF-γ sample by the end of the experiment, whereas remaining susceptible (S) calves had no positive INF-γ samples. R_0_^IG^ was the estimate of the number of new INF-γ positive infections that would result from the introduction of 1 INF-γ positive calf to the susceptible population. For both definitions, IN calves started the trial with infected status (I), and CE calves were considered susceptible (S), as all IN calves met the INF-γ and tissue culture criteria for categorization as infected calves. By the end of the trial, outcomes for the number of cases (C) in a pen could be 0, 1 or 2 where the number of susceptible calves will be 2, 1, or 0 (respectively) depending on the extent of transmission, and the formula for the probability of each outcome is as follows [27]:

$$p\left[ {{\text{S}} = 2} \right] = {\text{p}}\left[ {{\text{C}} = 0} \right] = \left( {\frac{4}{{2R_{0} + 4}}} \right)^{2}$$

$$p\left[ {{\text{S}} = 1} \right] = {\text{p}}\left[ {{\text{C}} = 1} \right] = \left( {\frac{4}{{2R_{0} + 4}} + \frac{4}{{R_{0} + 4}}} \right)\left( {\frac{{2R_{0} }}{{2R_{0} + 4}} + \frac{4}{{R_{0} + 4}}} \right)^{2}$$

$$p\left[ {{\text{S}} = 0} \right] = {\text{p}}\left[ {{\text{C}} = 2} \right] = 1 - \left( {\left( {p\left[ {{\text{S}} = 2} \right]} \right) + \left( {p\left[ {{\text{S}} = 1} \right]} \right)} \right)$$

These probabilities for each single experiment can be combined assuming that the experiments are independent by using the multinomial distribution with the 3 p values and the multinomial total being 17.

Thus, R_0_ can be derived analytically by the maximum likelihood estimate (MLE) given these probabilities for the definition of tissue infection (R_0_^T^) or INF-γ infection (R_0_^IG^) [27, 47].

## Results

### Detection of infection

Descriptive results were presented more extensively in the previous report [14]. All IN and CE calves had MAP-culture positive fecal samples at various times during group housing; however, CE calves ceased shedding after individual housing (Table 1). All IN calves and 5/14 CE calves had positive INF-γ samples, whereas all IN calves and 7/14 CE calves had at least 1 MAP culture-positive tissue sample (Table 1). All control calves tested positive for MAP in feces on day 21 of group housing, with 1 calf having an additional positive fecal sample on day 56. Positive fecal samples among control calves were likely due to contamination during laboratory processing and not fecal shedding, as all control animals tested positive on the same day. All control calves had negative tissue results and negative INF-γ results.

**Table 1:** **MAP culture results detected by F57-specific qPCR for fecal and tissue samples and INF-γ detection from whole blood samples**

Pen	Calf ID	IN:CE	MAP detection with F57 qPCR following culture of individual fecal samples														
			Days following group housing														
			0	3	5	7	10	12	14	17	19	21	24	26	28	31	33
1	1	IN	I		C	I		C		C			C	I	I		C
1	2	IN				C	I	I		C	I		C	I	I	I	I
1	15	CE								C	I						
1	16	CE									C						
2	3	IN		C	I	I			C	I	I	I	I	I	I		C
2	4	IN	I		C			C	I	I	I	I	I	I	I		C
2	17	CE				C		C	I							C	I
2	18	CE						C						C			C
3	5	IN					C		C	I		C	I	I	I	I	I
3	6	IN							C	I	I	I	I	I	I	I	I
3	19	CE						C	I								
3	20	CE							C						C		C
4	7	IN	I			C	I				C	I	I		C		
4	8	IN	I	I		C	I	I	I	I	I	I	I	I	I	I	I
4	21	CE												C		C	I
4	22	CE			C				C					C		C	
5	9	IN				C	I			C	I	I			C	I	I
5	10	IN				C		C	I	I	I	I	I	I	I	I	I
5	23	CE														C	
5	24	CE				C							C			C	
6	11	IN						C		C		C				C	
6	12	IN	I						C	C		C	I				C
6	25	CE									C					C	
6	26	CE													C		
7	13	IN		C				C			C	I	I		C		C
7	14	IN			C	I			C			C	I	I	I		C
7	27	CE					C			C			C			C	
7	28	CE									C		C				

Pen	Calf ID	IN:CE	MAP detection with F57 qPCR following culture of individual fecal samples														
			Days following group housing														
			35	38	40	42	45	47	49	52	54	56	59	61	63	66	68
1	1	IN	I	I	I	I	I	I	I	I	I						
1	2	IN		C	I	I	I		C	I	I	I		C			
1	15	CE	C	I			C		C								
1	16	CE		C	I	I	I						C				
2	3	IN	I	I	I	I	I	I	I							C	
2	4	IN	I	I	I	I		C	I		C	I	I	I	I		
2	17	CE	I	I	I											C	
2	18	CE	I													C	
3	5	IN	I	I	I	I	I	I	I	I	I	I	I	I	I	I	I
3	6	IN	I	I	I	I	I	I	I								
3	19	CE			C												
3	20	CE			C										C		
4	7	IN	C	I	I						C		C				
4	8	IN	I	I	I							C					
4	21	CE			C						C						
4	22	CE															
5	9	IN			C	I	I	I	I	I	I	I		C			
5	10	IN	I	I	I	I		C	I	I	I	I		C	I	I	I
5	23	CE				C											
5	24	CE											C				
6	11	IN	C						C				C				
6	12	IN	I			C		C	I	I	I				C		
6	25	CE								C							
6	26	CE															
7	13	IN	I	I	I	I		C	I	I	I						
7	14	IN	I	I				C			C				C		
7	27	CE		C									C				
7	28	CE													C		

Pen	Calf ID	IN:CE	MAP detection with F57 qPCR following culture of individual fecal samples													INF-ƴ	Tissue culture
			Days following group housing														
			70	73	75	77	80	82	84	*87*	*89*	*91*	*94*	*96*	*101*		
1	1	IN														+	+
1	2	IN														+	+
1	15	CE														−	+
1	16	CE							C							−	−
2	3	IN														+	+
2	4	IN														+	+
2	17	CE				C										+	+
2	18	CE								C						+	+
3	5	IN	I	I		C										+	+
3	6	IN		C												+	+
3	19	CE														−	+
3	20	CE							C							+	+
4	7	IN														+	+
4	8	IN														+	+
4	21	CE														−	+
4	22	CE		C												+	−
5	9	IN														+	+
5	10	IN	I		C	I				I						+	+
5	23	CE														+	−
5	24	CE														−	−
6	11	IN														+	+
6	12	IN	C													+	+
6	25	CE				C										−	−
6	26	CE	C						C							−	+
7	13	IN				C										+	+
7	14	IN														+	+
7	27	CE														−	−
7	28	CE						C								−	−

“I” indicate an infectious animal and “C” indicates a new shedding event. CE calves were individually housed 87 days following group housing (italic).

*CE* contact exposed, *IN* inoculated.

### GLM

There was no difference in infectiousness (ability to cause shedding in contact exposed calves) between CE and IN calves (C3 = 0), environmental or direct oral transmission (C4 = 0) and thus also not for the interaction of the 2 variables. Susceptibility to begin shedding was different for IN and CE calves (C1 ≠ 0), likely due to the difference in exposure (IN vs CE); therefore, the contrast was included as an explanatory variable in the final GLM model (*p* < 0.001). Explanatory variables also included in the final model were number of days following group housing date (day), the interaction between housing date and inoculation status and the intercept. All estimates of transmission rate parameters were calculated for the beginning of the experiment at “day” = 0; therefore, the interaction between “day” and susceptibility status was not included in the final model (Table 2). When the interaction was included in the model, there was no significant difference in how the model fit the data and the difference in the coefficients are shown (Table 2). No coefficients of the explanatory variables changed more than 11%; therefore, it was concluded that no confounding was present.

**Table 2:** **Estimates for coefficients and their 95% confidence for fecal shedding of** ***Mycobacterium avium*** **subsp.** ***paratuberculosis*** in final General Linearized Model (GLM) with and without the interaction term for start day*susceptibility status

Model	Model coefficient (code)	Estimate (day^−1^)	*p* value	95% confidence interval	AIC value for model fit
Final model with no interaction between susceptibility and day					530.67
	Susceptibility status (C1)	1.41	< 0.001	1.05, 1.77	
	Time since group housing (C2)	− 0.027	< 0.001	− 0.035, − 0.019	
	Infectiousness of contact exposed calves (C0)	− 1.84	< 0.001	− 2.21, − 1.47	
Model including interaction between susceptibility and day					527.86
	Susceptibility status (C1)	2.08	< 0.001	1.37,2.79	
	Time since group housing (C2)	− 0.014	0.033	− 0.028, − 0.001	
	Interaction (C5)	− 0.019	0.028	− 0.036, − 0.002	
	Infectiousness of contact exposed calves (C0)	− 2.26	< 0.001	− 2.82, − 1.71	

As IN and CE calves had different infectious intervals, R_0_ had to be estimated separately for these 2 groups of calves. The transmission rate parameter β_CE_CE_ was estimated as 0.158 per day (0.109, 0.230). The average infectious period for CE calves was 2.91 (0.98, 6.08) days. Therefore, the equation to calculate R_0_^CE^ is as follows:

$${\text{R}}_{0} = 0.158 *2.91 *(1 -\upsigma)^{ - 1}$$

Based on the GLM, the estimated basic reproduction value for CE calves (R_0_^CE^) was 3.24 (1.41, 7.41). Thus, the transmission of fecal shedding events among calves by themselves without the effect of inoculation is significantly above one, i.e. MAP is transmitted among calves.

For IN calves, the transmission rate parameter β_IN_IN_ was 0.649 per day (0.437, 0.965) and the average infectious period was 7.49 (0, 25.3) days; therefore, the equation to calculate the reproduction value for IN calves was as follows:

$${\text{R}}_{0} = 0.649 *7.491 *(1 -\upsigma)^{ - 1}$$

The estimated reproduction value for IN calves R_0_^IN^ was 24.6 (4.57, 133.3). Note that as inoculated calves were only inoculated at the beginning of the trial, the estimate is not in itself a reproduction ratio of a population of calves, but rather reflects the initial susceptibility to begin shedding of calves exposed in a different way (inoculation) then by calf-to-calf transmission.

### FS model

When quantifying transmission based on the INF-γ definition of MAP infection, in 3 pens no susceptible (S) calves became infected (I), in 2 pens only 1 of the 2 susceptible (S) calves became infected (I) and in 1 pen, all susceptible (S) calves became infected (I). Therefore, the estimated reproduction value for defining infected calves as infected based on INF-γ (R_0_^IG^) was 0.90 (0.24, 2.59).

When quantifying transmission based on being tissue culture-positive as a definition of MAP infection, in 2 pens none of the susceptible (S) calves became infected (I), in 3 pens, 1 susceptible calf (S) became infected and in 2 pens all susceptible (S) calves became infected (I). Therefore, the estimated reproduction value for defining infected calves based on tissue (R_0_^T^) was 1.36 (0.45, 3.94).

## Discussion

Quantification of transmission of MAP infection based on fecal shedding and accumulation of environmental contamination indicated that one CE calf that has a new shedding event within a completely susceptible population of calves in a clean environment could cause approximately 3 calves to begin shedding (R_0_^CE^ = 3.24; 1.41, 7.41). This estimate represents MAP transmission for calves in a herd entering a clean environment as it starts from a “naturally” infected calf. Using MAP-positive tissue culture, or cellular immune response to MAP, one infectious calf would cause approximately one CE calf to become infected in group housing (R_0_^T^ = 1.36; and R_0_^IG^ = 0.90, respectively). Thus, the R_0_ value for MAP transmission among naturally infected calves lies between 0.9 and 3.24 depending on the definition of infection (fecal shedding, INF-γ response, or tissue infection). Our study indicated that transmission of MAP between group-housed dairy calves occurred (R > 1) and that potential shedding outbreaks may occur. When including the interaction between “day” and susceptibility based on exposure status, the transmission estimate for CE calves decreases, indicating that over time infectiousness or susceptibility to begin shedding decreases. However, this change may be due to several extraneous variables specific to the trial and the environmental contamination of MAP (bed pack, addition of clean material, cleaning of environment, etc.) as well as the possibility that CE calves are already infected and shedding events are due to intermittent active shedding rather than the result of penmates shedding. Therefore, the model without the interaction provides a better estimate of the transmission estimate for group-housed calves entering a clean environment (corresponding to the beginning of the trial, day = 0).

An important factor to take into consideration is that duration of fecal shedding was determined by the number of days that fecal samples tested positive for MAP. It is possible that due to intermittent shedding, detection of a positive shedding event may be influenced by these patterns [14] and affect the length of shedding event (or infectious period) in the current model. However, fecal samples were collected more frequently than previously reported [12, 48] and cultured, we expected to have captured more shedding events and more accurately estimated the infectious period. Additionally, the estimate of the transmission rate parameter beta is based on the same definition of infectiousness for calves. Therefore, although the interval of shedding may be underestimated, there is no effect on the reproduction ratio.

That the R_0_^CE^ value for fecal shedding among calves was higher than previously described [16, 17], this may be due to the fact that new shedding events were measured and the difference between active shedding and passive shedding cannot be differentiated. Although these types of shedding are not discernable, evidence of fecal shedding is typically assumed to be an indicator of infection and any shedding event is used to identify infected animals [16, 17]. Additionally, environmental contamination was taken into consideration in the current study. All calves shed MAP into the group pen and due to the resilience of MAP, the presence of MAP in this environment remained a source of infection for a prolonged interval. Presence of MAP in the environment, especially resulting from a high herd prevalence of MAP, has a large impact on fecal–oral transmission from cows to calves [49–51]. In the analysis of our experiments, only when environmental contamination with MAP was taken into consideration did the model better capture transmission of fecal shedding between calves, based on AIC values. Both IN and CE calves shed MAP into the environment with no difference between infectivity rates (β_IN_ = β_CE_); however, there was a difference in the IN or CE calves’ susceptibilities to begin shedding and duration of shedding events. The IN calves were more likely to begin shedding and had longer shedding events, leading to a higher point estimate R_0_ value (24.6) than CE calves (3.2). Indirect transmission of infection (indicated by positive tissue and immune responses) that may occur through the environment is likely to have an impact on calf-to-calf transmission, especially considering the large amount of intermittent shedding that occurs within these animals and opportunities to miss detection of shedding events. It should be taken into consideration that the R_0_^CE^ may be an overestimation of new cases of shedding, as intermittent shedding could not be taken into consideration and each new shedding event was considered a new case of shedding rather than a result from the original exposure. However, it should be considered that all CE calves ceased to shed following individual housing in a clean environment. With both IN and CE calves, it is possible that individuals were already infected and shedding events were due to intermittent shedding, especially among IN calves. However, it is not possible to differentiate which events were due to intermittent shedding, new infections and active shedding, or passive shedding. That all IN and CE animals shed at least twice during group-housing, FS estimation based on fecal samples would result in an infinite R_0_ value; therefore, the GLM model based on new shedding events was deemed most appropriate.

One of the difficulties associated with MAP is that an animal can be “infectious” and/or “infected”; therefore a GLM model was used to identify “infectious” calves, whereas the FS model was used to identify “infected” calves and transmission of infected status [52]. Although fecal shedding may not be a true indication of MAP infection and could be the result of either passive or active shedding, the evidence that 1 CE calf might cause 3 additional calves in the population to have shedding events has important implications for JD control programs. Any pass-through of infected material (pass-through shedding) has potential to cause infection within the shedding calf; therefore, new shedding events may be of importance to control programs, as these calves may develop an active infection. Although all calves had positive fecal shedding detected throughout the study, not all had positive tissue samples. Transmission of fecal shedding among calves may be one explanation for the low, consistent prevalence and maintenance of MAP infections on farm. Of all CE calves that were fecal shedding, half (7/14) had culture-positive tissue samples and the estimated R_0_^T^ value for “transmission” of MAP-positive tissue samples from 1 CE calf to a susceptible calf was 1.36. However, this was not significantly larger than 1. Calves that are tissue-positive, may cause 1 other calf in the group pen to become tissue-positive through fecal shedding, potentially leading to infection and symptoms later in life; however, this is only part of all MAP transmission dynamics that occur on farm. Additionally, it should be taken into consideration that testing of tissue samples in non-clinical calves may lead to a high number of false-negative samples, due to low MAP concentrations in tissue and few samples sites that are positive, which would lead to an underestimation of the number of infected calves [13, 53–55]. The tendency for calves to shed MAP, but only some to have detectable positive tissue samples indicating infection, may be one explanation for the low, consistent prevalence and maintenance of MAP infections on farm.

Epidemiological models are a useful tool when testing a hypothesis in the field is difficult or improbable. This is the case with MAP, as infections are slow progressing with a long latent period, susceptibilities and infectivities may vary between age groups, there are low sensitivities and specificities of diagnostic tests and not all transmission routes (including the environmental impact) are clearly understood [28]. These models are simplified representations based on current knowledge designed to investigate specific hypotheses such as: MAP transmission dynamics in a herd [32, 56], impact of control programs [33, 35], predicting fadeout and persistence of disease [19, 29] and effects of infectious young stock on disease control [20]. Outcomes of each of these models rely on the availability of knowledge regarding the underlying mechanisms of the disease, as well as assumptions made to fill in knowledge gaps and the modelling objective to answer the question at hand [28]. Due to contradictory findings regarding the impact of calf-to-calf transmission [16, 19, 20, 29, 56], inconsistent values are used to model the impact of infection and transmission rates of young stock and subsequent herd transmission dynamics. Note that in the earlier experimental quantification of transmission [16] the estimate of R was below 1 and we now know that this could have been caused by not taking the environment into account in the estimation, because the time to first contact infection underestimates the transmission rate parameter as transmission increases after being placed in a clean environment. The current study provides R_0_ values for calf-to-calf transmission that can be used to better model transmission dynamics on farm, with potential to examine different infectious abilities. The fecal shedding R_0_ value demonstrates occurrence of new shedding events caused by infectious calves and the contaminated environment in group-housed pens; however, this value did not necessarily represent infections that will persist over time. Based on INF-γ or tissue results, a more conservative estimate for persistent infections in calves could be obtained. Although these different R_0_ values correspond to different parameters of a model and different definitions of infection, these results may enable better, more accurate modelling of infection and transmission of MAP within dairy farms, leading to more effective control and prevention programs.

Currently, control programs focus on decreasing transmission to susceptible calves from infectious dams by separating young stock soon after birth, based on the assumption calves will not transmit infections to each other [10]. However, results from this study indicated that shedding of MAP may be more easily transmitted to other pen mates than previously assumed, not only from direct contact, but potentially more importantly, due to environmental contamination caused by shedding animals. Individual calf housing may be one solution to the risk of calf-to-calf transmission; however, this method of calf housing may not be possible for all herds, due to increased labour costs [57], transition to automated feeding systems [58], decreased calf welfare [59–61] and the need for effective cleaning between successive calves. A second solution would be regular and vigorous maintenance of a clean group-housed environment, in which contaminated material is removed and replaced with new bedding and base, and sides of the enclosure are disinfected. Additionally, young stock should be considered for inclusion of MAP testing in a herd testing program, as up to 2% of young stock on positive farms have been identified positively for MAP shedding [15]. Animals with positive fecal samples, or whose environment tests are positive for MAP, should be immediately removed from group-housing and monitored/tested in the future to determine infection status. Although not all infected or infectious animals may be detected due to intermittent shedding and lack of sensitivity in immune tests, this would be an added measure that may decrease MAP prevalence on dairy farms.

Results from this research indicate a potential for group-housed calves to cause shedding among penmates, leading sometimes to more extensive infection as evidenced by tissue and immune responses; however, consideration should be made for parameters of the experimental situation when applying results to the field. Future research is required regarding calf-transmission that occurs on farms and effects of a contaminated environment, as positive young stock have been detected in positive herds [15]. Ideally, a longitudinal study on infected farms should be conducted in which fecal samples, environmental samples and blood samples are collected regularly from all penmates that are group-housed, beginning at birth, for the entire duration of group housing. These longitudinal studies will lead to a better understanding of transmission rates and infection status that may occur due to direct contact and/or environmental contamination that occurs within a commercial dairy herd.

In conclusion, in this study, transmission of MAP among group-housed calves was quantified using GLM and FS modelling, based on various definitions of infectiousness or infection. The GLM model was based on the propensity of new shedding events to occur, whereas the FS model was based on tissue or immune responses as a definition of infection. Based on the definition of infection or infectious, the R_0_ value for MAP was between 0.9 and 3.24. Using the GLM model of fecal shedding over time and an SIS model, the R_0_^CE^ for an infectious contact-exposed animal was estimated to be 3.24. However, this model only considered fecal shedding and therefore was not a perfect indication of infection among calves. Alternatively, FS models using positive tissue and immune responses as a definition of infection had R_0_ values equal to 1.36 and 0.9, respectively. Together, this study provided evidence that transmission of MAP infection among group-housed calves was not only present, but may be a contributing factor to maintenance of infections within a herd and therefore, should be seriously considered in future Johne’s disease prevention and control programs.

## Abbreviations

C: case
CE: contact-exposed
FS: final size
GLM: general linear method
I: infectious
IN: inoculated
JD: Johne’s disease
MAP: *Mycobacterium avium* subspecies *paratuberculosis*
S: susceptible
R_0_^CE^: the number of calves to have a new fecal-shedding event caused by one fecal shedding CE calf
R_0_^IN^: the number of calves to have a new fecal-shedding event caused by one fecal shedding IN calf
R_0_^IG^: the number of new INF-γ positive calves caused by one INF-γ positive calf
R_0_^T^: the number of new tissue positive calves caused by one tissue positive calf

## References

[1] Wolf R, Barkema HW, De Buck J, Slomp M, Flaig J, Haupstein D, Pickel C, Orsel K (2014). High herd-level prevalence of *Mycobacterium avium* subspecies *paratuberculosis* in Western Canadian dairy farms, based on environmental sampling. J Dairy Sci.

[2] McKenna SL, Keefe GP, Tiwari A, VanLeeuwen JA, Barkema HW (2006). Johne’s disease in Canada Part II: disease impacts, risk factors, and control programs for dairy producers. Can Vet J.

[3] Cho J, Tauer LW, Schukken YH, Smith RL, Lu Z, Gröhn YT (2013). Cost-effective control strategies for Johne’s disease in dairy herds. Can J Agric Econ.

[4] Collins MT, Eggleston V, Manning EJ (2010). Successful control of Johne’s disease in nine dairy herds: results of a six-year field trial. J Dairy Sci.

[5] Koets A, Hoek A, Langelaar M, Overdijk M, Santema W, Franken P, Eden W, Rutten V (2006). Mycobacterial 70 kD heat-shock protein is an effective subunit vaccine against bovine paratuberculosis. Vaccine.

[6] Kalis CH, Hesselink JW, Barkema HW, Collins MT (2001). Use of long-term vaccination with a killed vaccine to prevent fecal shedding of *Mycobacterium avium* subsp *paratuberculosis* in dairy herds. Am J Vet Res.

[7] Tiwari A, VanLeeuwen JA, McKenna SL, Keefe GP, Barkema HW (2006). Johne’s disease in Canada Part I: clinical symptoms, pathophysiology, diagnosis, and prevalence in dairy herds. Can Vet J.

[8] Harris NB, Barletta RG (2001). *Mycobacterium avium* subsp. *paratuberculosis* in veterinary medicine. Clin Microbiol Rev.

[9] Slater N, Mitchell RM, Whitlock RH, Fyock T, Pradhan AK, Knupfer E, Schukken YH, Louzoun Y (2016). Impact of the shedding level on transmission of persistent infections in *Mycobacterium avium* subspecies *paratuberculosis* (MAP). Vet Res.

[10] Garry F (2011). Control of paratuberculosis in dairy herds. Vet Clin North Am Food Anim Pract.

[11] Whitlock RH, Behr MA, Collins DM (2010). Paratuberculosis control measure in the USA. Paratuberculosis—organism, disease and control.

[12] Mortier R, Barkema HW, Orsel K, Wolf R, De Buck J (2014). Shedding patterns of dairy calves experimentally infected with *Mycobacterium avium* subspecies *paratuberculosis*. Vet Res.

[13] Mortier R, Barkema HW, Bystrom J, Illanes O, Orsel K, Wolf R, Atkins G, De Buck J (2013). Evaluation of age-dependent susceptibility in calves infected with two doses of *Mycobacterium avium* subspecies *paratuberculosis* using pathology and tissue culture. Vet Res.

[14] Corbett CS, De Buck J, Orsel K, Barkema HW (2017). Fecal shedding and tissue infections demonstrate transmission of *Mycobacterium avium* subsp. *paratuberculosis* in group-housed dairy calves. Vet Res.

[15] Wolf R, Orsel K, De Buck J, Barkema HW (2015). Calves shedding *Mycobacterium avium* subspecies *paratuberculosis* are common on infected dairy farms. Vet Res.

[16] van Roermund HJ, Bakker D, Willemsen PT, de Jong MC (2007). Horizontal transmission of *Mycobacterium avium* subsp. *paratuberculosis* in cattle in an experimental setting: calves can transmit the infection to other calves. Vet Microbiol.

[17] Santema WJ, Poot J, Segers RP, Van den Hoff DJ, Rutten VP, Koets AP (2012). Early infection dynamics after experimental challenge with *Mycobacterium avium* subspecies *paratuberculosis* in calves reveal limited calf-to-calf transmission and no impact of Hsp70 vaccination. Vaccine.

[18] Mitchell RM, Medley GF, Collins MT, Schukken YH (2012). A meta-analysis of the effect of dose and age at exposure on shedding of *Mycobacterium avium* subspecies *paratuberculosis* (MAP) in experimentally infected calves and cows. Epidemiol Infect.

[19] Marcé C, Ezanno P, Seegers H, Pfeiffer DU, Fourichon C (2011). Predicting fadeout versus persistence of paratuberculosis in a dairy cattle herd for management and control purposes: a modelling study. Vet Res.

[20] Weber MF, Groenendaal H (2012). Effects of infectious young stock on results of certification, surveillance and control programmes for paratuberculosis in dairy herds. Vet Microbiol.

[21] van Roermund HJ, Vos AM, De Jong MC (2002) Within herd transmission of paratuberculosis and the possible role of infectious calves. In: Proceedings of the 7th international colloquium paratuberculosis; Bilbao, Spain; pp 368–370

[22] Bravo de Rueda C, de Jong MC, Eble PL, Dekker A (2015). Quantification of transmission of foot-and-mouth disease virus caused by an environment contaminated with secretions and excretions from infected calves. Vet Res.

[23] Velthuis AG, De Jong MC, De Bree J (2007). Comparing methods to quantify experimental transmission of infectious agents. Math Biosci.

[24] Broens EM, Espinosa-Gongora C, Graat EAM, Vendrig N, Van Der Wolf PJ, Guarabassi L, Butaye P, Neilsen JP, De Jong MCM, Van De Giessen AW (2012). Longitudinal study on transmission of MRSA CC398 within pig herds. BMC Vet Res.

[25] Huijbers PM, Graat EA, van Hoek AH, Veenman C, de Jong MC, van Duijkeren E (2016). Transmission dynamics of extended-spectrum beta-lactamase and AmpC beta-lactamase-producing *Escherichia coli* in a broiler flock without antibiotic use. Prev Vet Med.

[26] Diekmann O, Heesterbeek JAP, Metz JAJ (1990). On the definition and the computation of the basic reproduction ratio R_0_ in models for infectious diseases in heterogeneous populations. J Math Biol.

[27] Velthuis AGJ, De Jong MCM, De Bree J, Nodelijk G, Van Boven M (2002). Quantification of transmission in one-to-one experiments. Epidemiol Infect.

[28] Marcé C, Ezanno P, Weber MF, Seegers H, Pfeiffer DU, Fourichon C (2010). Invited review: modeling within-herd transmission of *Mycobacterium avium* subspecies *paratuberculosis* in dairy cattle: a review. J Dairy Sci.

[29] Mitchell RM, Whitlock RH, Stehman SM, Benedictus A, Chapagain PP, Grohn YT, Schukken YH (2008). Simulation modeling to evaluate the persistence of *Mycobacterium avium* subsp. *paratuberculosis* (MAP) on commercial dairy farms in the United States. Prev Vet Med.

[30] Pouillot R, Dufour B, Durand B (2004). A deterministic and stochastic simulation model for intra-herd paratuberculosis transmission. Vet Res.

[31] Humphry RW, Stott AW, Adams C, Gunn GJ (2006). A model of the relationship between the epidemiology of Johne’s disease and the environment in suckler-beef herds. Vet J.

[32] Al-Mamun MA, Smith RL, Schukken YH, Grohn YT (2016). Modeling of *Mycobacterium avium* subsp. *paratuberculosis* dynamics in a dairy herd: an individual based approach. J Theor Biol.

[33] Smith RL, Al-Mamun MA, Gröhn YT (2017). Economic consequences of paratuberculosis control in dairy cattle: a stochastic modeling study. Prev Vet Med.

[34] Collins MT, Morgan IR (1991). Epidemiological model of paratuberculosis in dairy cattle. Prev Vet Med.

[35] Kudahl AB, Ostergaard S, Sorensen JT, Nielsen SS (2007). A stochastic model simulating paratuberculosis in a dairy herd. Prev Vet Med.

[36] Barkema HW, Orsel K, Nielsen SS, Koets AP, Rutten V, Bannantine JP, Keefe GP, Kelton DF, Wells SJ, Whittington RJ, Mackintosh CG, Manning EJ, Weber MF, Heuer C, Forde TL, Ritter C, Roche S, Corbett CS, Wolf R, Griebel PJ, Kastelic JP, De Buck J (2018). Knowledge gaps that hamper prevention and control of *Mycobacterium avium* subspecies *paratuberculosis* infection. Transbound Emerg Dis.

[37] Hines ME, Stabel JR, Sweeney RW, Griffin F, Talaat AM, Bakker D, Benedictus G, Davis WC, de Lisle GW, Gardner IA, Juste RA, Kapur V, Koets A, McNair J, Pruitt G, Whitlock RH (2007). Experimental challenge models for Johne’s disease: a review and proposed international guidelines. Vet Microbiol.

[38] Sweeney RW, Uzonna J, Whitlock RH, Habecker PL, Chilton P, Scott P (2006). Tissue predilection sites and effect of dose on *Mycobacterium avium* subs. *paratuberculosis* organism recovery in a short-term bovine experimental oral infection model. Res Vet Sci.

[39] Mortier RA, Barkema HW, De Buck J (2015). Susceptibility to and diagnosis of *Mycobacterium avium* subspecies *paratuberculosis* infection in dairy calves: a review. Prev Vet Med.

[40] Forde T, Kutz S, De Buck J, Warren A, Ruckstuhl K, Pybus M, Orsel K (2012). Occurence, diagnosis, and strain typing of *Mycobacterium avium* subsp. *paratuberculosis* infection in Rocky Mountain bighorn sheep (*Ovis canadensis canadensis*) in southwest Alberta. J Wildlife Dis.

[41] Slana I, Kralik P, Kralova A, Pavlik I (2008). On-farm spread of *Mycobacterium avium* subsp. *paratuberculosis* in raw milk studied by IS900 and F57 competitive real time quantitative PCR and culture examination. Int J Food Microbiol.

[42] Mortier RA, Barkema HW, Wilson TA, Sajobi TT, Wolf R, De Buck J (2014). Dose-dependent interferon-gamma release in dairy calves experimentally infected with *Mycobacterium avium* subspecies *paratuberculosis*. Vet Immunol Immunopathol.

[43] Kalis CHJ, Collins MT, Hesselink JW, Barkema HW (2003). Specificity of two tests for the early diagnosis of bovine paratuberculosis based on cell-mediated immunity: the Johnin skin test and the gamma interferon assay. Vet Microbiol.

[44] Velthuis AGJ, De Jong MCM, Kamp EM, Stockhofe N, Verheijden JHM (2003). Design and analysis of an *Actinobacillus pleuropneumoniae* transmission experiment. Prev Vet Med.

[45] de Jong MCM, Diekmann O, Heesterbeek H, Mollison D (1995). How does transmission of infection depend on population size?. Epidemic models: their structure and relation to data.

[46] De Silva KR, Eda S, Lenhart S (2017). Modeling environmental transmission of MAP infection in dairy cows. Math Biosci Eng.

[47] Kroese AH, De Jong MCM (2001) Design and analysis of transmission experiments. In: Meeting for the society for veterinary epidemiology and preventative medicine. society for veterinary epidemiology and preventative medicine; pp xxi–xxxvii

[48] Munster P, Volkel I, Wemheuer W, Schwarz D, Doring S, Czerny CP (2013). A longitudinal study to characterize the distribution patterns of *Mycobacterium avium* ssp. *paratuberculosis* in semen, blood and faeces of a naturally infected bull by IS 900 semi-nested and quantitative real-time PCR. Transbound Emerg Dis.

[49] Windsor PA, Whittington RJ (2010). Evidence for age susceptibility of cattle to Johne’s disease. Vet J.

[50] Kralik P, Pribylova-Dziedzinska R, Kralova A, Kovarcik K, Slana I (2014). Evidence of passive faecal shedding of *Mycobacterium avium* subsp. *paratuberculosis* in a Limousin cattle herd. Vet J.

[51] Wolf R, Barkema HW, De Buck J, Orsel K (2016). Dairy farms testing positive for *Mycobacterium avium* ssp. *paratuberculosis* have poorer hygiene practices and are less cautious when purchasing cattle than test-negative herds. J Dairy Sci.

[52] Nielsen SS, Toft N (2006). Age-specific characteristics of ELISA and fecal culture for purpose-specific testing for paratuberculosis. J Dairy Sci.

[53] Koets AP, Eda S, Sreevatsan S (2015). The within host dynamics of *Mycobacterium avium* ssp. *paratuberculosis* infection in cattle: where time and place matter. Vet Res.

[54] Buergelt CD, Hall C, McEntee K, Duncan JR (1978). Pathological evaluation of paratuberculosis in naturally infected cattle. Vet Pathol.

[55] McDonald WL, Ridge SE, Hope AF, Condron RJ (1999). Evaluation of diagnostic tests for Johne’s disease in young cattle. Aust Vet J.

[56] Marcé C, Ezanno P, Seegers H, Pfeiffer DU, Fourichon C (2011). Within-herd contact structure and transmission of *Mycobacterium avium* subspecies *paratuberculosis* in a persistently infected dairy cattle herd. Prev Vet Med.

[57] Broom DM, Leaver JD (1978). Effects of group-rearing or partial isolation on later social behaviour of calves. Anim Behav.

[58] Barkema HW, von Keyserlingk MA, Kastelic JP, Lam TJ, Luby C, Roy JP, LeBlanc SJ, Keefe GP, Kelton DF (2015). Invited review: changes in the dairy industry affecting dairy cattle health and welfare. J Dairy Sci.

[59] Chua B, Coenen E, van Delen J, Weary DM (2002). Effects of pair versus individual housing on the behavior and perfomance of dairy calves. J Dairy Sci.

[60] Gaillard C, Meagher RK, von Keyserlingk MA, Weary DM (2014). Social housing improves dairy calves’ performance in two cognitive tests. PLoS ONE.

[61] Jensen MB, Larsen LE (2014). Effects of level of social contact on dairy calf behavior and health. J Dairy Sci.

